# Abundances of Tetracycline, Sulphonamide and Beta-Lactam Antibiotic Resistance Genes in Conventional Wastewater Treatment Plants (WWTPs) with Different Waste Load

**DOI:** 10.1371/journal.pone.0103705

**Published:** 2014-08-01

**Authors:** Mailis Laht, Antti Karkman, Veiko Voolaid, Christian Ritz, Tanel Tenson, Marko Virta, Veljo Kisand

**Affiliations:** 1 Institute of Technology, University of Tartu, Tartu, Estonia; 2 Department of Food and Environmental Sciences, University of Helsinki, Helsinki, Finland; 3 Department of Nutrition, Exercise and Sports, Faculty of Science, University of Copenhagen, Copenhagen, Denmark; University of Nordland, Norway

## Abstract

Antibiotics and antibiotic resistant bacteria enter wastewater treatment plants (WWTPs), an environment where resistance genes can potentially spread and exchange between microbes. Several antibiotic resistance genes (ARGs) were quantified using qPCR in three WWTPs of decreasing capacity located in Helsinki, Tallinn, and Tartu, respectively: sulphonamide resistance genes (*sul1* and *sul2*), tetracycline resistance genes (*tetM* and *tetC*), and resistance genes for extended spectrum beta-lactams (*bla_oxa-58_, bla_shv-34_, and bla_ctx-m-32_*). To avoid inconsistencies among qPCR assays we normalised the ARG abundances with 16S rRNA gene abundances while assessing if the respective genes increased or decreased during treatment. ARGs were detected in most samples; *sul1, sul2,* and *tetM* were detected in all samples. Statistically significant differences (adjusted p<0.01) between the inflow and effluent were detected in only four cases. Effluent values for *bla_oxa-58_* and *tetC* decreased in the two larger plants while *tetM* decreased in the medium-sized plant. Only *bla_shv-34_* increased in the effluent from the medium-sized plant. In all other cases the purification process caused no significant change in the relative abundance of resistance genes, while the raw abundances fell by several orders of magnitude. Standard water quality variables (biological oxygen demand, total phosphorus and nitrogen, etc.) were weakly related or unrelated to the relative abundance of resistance genes. Based on our results we conclude that there is neither considerable enrichment nor purification of antibiotic resistance genes in studied conventional WWTPs.

## Introduction

Antibiotic resistance (AR) has become a worldwide problem, making infectious diseases more resilient thus making treatment more difficult and costly [1]. AR is not confined to hospital environments, and is able to spread between both human dominated and natural environments. Increased amounts of antibiotic residues [2]–[4] and antibiotic resistant bacteria (ARB) are found in human-related environments such as agricultural settings (e.g. farms, soil etc.) [5], [6] and surface-, drinking- and wastewaters [7]–[11]. Wastewater treatment plants (WWTPs) receive sewage from various sources, including hospitals and households which are both important sources of antibiotics and their residues [12]–[14]. and antibiotic resistant bacteria (ARB) [15]–[17]. The presence of antibiotics and antibiotic residues [18]. ARB, and antibiotic resistance genes (ARG) have been confirmed in many WWTPs [19]–[23]. Bacteria from various environments, including human, soil, and activated sludge, are mixed in WWTPs and therefore these facilities are considered to be important “hot-spots” for AR and spread of resistance genes [18], [24]–[26]. The presence of antibiotics, ARB, and ARG in the same setting creates an environment that selects for AR and provides an opportunity for genetic material housing ARGs to transfer between bacterial species via horizontal gene transfer [13], [21], [24], [25], [27], [28]. In a metagenomic study of plasmids it was shown that numerous medically relevant ARG can be found in WWTPs (140 ARGs in a single WWTP and 123 in the effluent water) [29]. Therefore, there is a concern that resistance genes will spread in the bacterial population and further into more natural environments less impacted by human activity [30]–[33].

Many studies of antibiotic resistant bacteria and resistance genes have used culture-based assays, which are biased towards specific cultivable pathogenic or environmental species [17], [19], [21], [23], [34]. Culture-dependent data does not reflect the real variability or the actual amount of resistance genes present in a given WWTP, so these studies normally characterize only a small subset of the total population [35], [36]. Surprisingly, only a limited number of studies have used quantitative methods to investigate resistance genes in the total communities in effluent waters [21], [33], [37]–[40]. The small number of quantitative studies could be one reason why the overall impact of WWTPs in spreading resistance to the environment has not yet been properly evaluated.

The current study was designed to investigate the role of three conventional WWTPs in the distribution of ARGs using culture-independent quantitative methods. We thus focus on quantitative whole community level measurements on the water-phase of the inflow and effluent of WWTPs which has not been studied using quantitative methods in sufficient detail to assess the impact of WWTPs on the distribution of ARGs. To quantify the number of ARGs we analysed the total DNA from wastewater and effluent samples using quantitative real-time PCR (qPCR). Our original hypothesis was that conventional WWTPs increase the relative abundance of ARGs during processing because they do not employ technologies that target the removal of genetic elements.

## Materials and Methods

### 1.1. Sample collection

Both raw wastewater and final effluent water samples were collected from three city WWTPs of decreasing capacity located in the Baltic Sea catchment area: large (Helsinki, Finland), medium (Tallinn, Estonia), and small (Tartu, Estonia). No specific permissions were required for sampling these locations, and the sampling was carried out in collaboration with each WWTP staff. The wastewater treatment technology employed in these three WWTPs is similar and typical of other facilities located in both Nordic (Finland, Sweden, Norway) and Eastern European (Estonia, Latvia, Lithuania, Poland) countries (EEA 2013). The WWTP in Helsinki is both the largest wastewater treatment plant in Finland and all Nordic countries; with about 0.8 million residents in the Helsinki metropolitan area. The majority of wastewater in Estonia is produced in Tallinn (∼350 000 residents) and Tartu (∼100 000). To abide by European law (91/271/EEC), the main steps of treatment are: primary treatment - mechanical treatment steps (sand, grit, fat and grease removal, pre-sedimentation); secondary treatment - biological treatment (activated sludge); followed by tertiary treatment - final deep purification using a combination of methods (chemical, mechanical and biological as in secondary sedimentation, bio-filters etc.). The WWTP effluent in both Helsinki and Tallinn is directed into the Baltic Sea while the WWTP effluent in Tartu flows into the Emajõgi River which forms part of the Baltic Sea catchment area. The main steps in the WWTP process and water-phase treatment are presented in Figure S1 in File S1.

Samples were collected over a one year period from December 2010 to December 2011 at five different time points, each representing a different season (four seasons; winter was sampled twice, Table S1A in File S1). In each sampling period three consecutive samples were taken on separate days at 1–3 day intervals; the exact sampling dates and monitored variables are given in Table S1 in File S1. Composite samples were taken, collected over 24 h periods, except in Tartu in the 2011 winter season when a grab sample was taken owing to technical problems (the automatic sampler was frozen because of extremely low temperatures).

### 1.2. Collection of total microbial community

The water samples were stored at 4°C pending filtration (within a couple of hours). Ten ml of influent water and 100 ml of effluent water were filtered through polycarbonate filters (pore size 0.22 µm, diameter 47 mm, GE Water & Process Technologies). For the last two time points at the Tallinn WWTP 200 ml of effluent water was filtered because a new treatment step (bio-filter) was added in September 2011.

### 1.3. DNA extraction

For the first three time points (Dec 2010; March 2011; June 2011) from the Helsinki WWTP, DNA was extracted from the samples using a MoBio PowerWater DNA isolation kit (MoBio Laboratories, Inc., CA, USA). For all other samples, the DNA was extracted using the modified bead beating and silica-membrane method (nucleic acid binding on to silica particles [41]. Method in brief: for lysis: 400 µl TE+50 µl lysozyme (50 mg/ml from egg yolk) was added to the filter and incubated at 37°C for 15 min. Fifty µl of 10% SDS and 500 µl lysis buffer BQ1 (NucleoSpin, Macherey-Nagel)+zirconium beads (0.1 mm diameter; burned at 500°C) were added and the samples were processed by a 5 min beating on a bead beater (Biospec products, Minibead beater). Thereafter, proteinase K was added followed by 15 min at 65°C with constant shaking. Five hundred µl of 96% ethanol was added and the whole volume was applied to commercial silica-membrane columns (NucleoSpin Macherey-Nagel). Finally, the total DNA was recovered in 50 µl of elution buffer BE (NucleoSpin Macherey-Nagel, 5 mM Tris/HCl pH 8.5). The concentration of extracted DNA was measured using a NanoDrop Spectrophotometer ND-1000 (absorption readings at 260 nm). The extracted DNA was stored at −20°C pending further analysis.

### 1.4. Detection and quantification of ARG copy number by qPCR

Seven resistance genes were surveyed: *sul1, sul2, tetM, tetC, bla_shv-34_, bla_ctx-m-32_*, and *bla_oxa-58_*. For the first three time point samples (Dec 2010; March 2011; June 2011) from the Helsinki WWTP (Assay 1), qPCR was performed using a Dynamo Flash SYBR Green qPCR kit (Thermo Scientific, Lithuania) and a 7300 Real-Time PCR system (Applied Biosystems, Foster City, CA, USA). The thermal cycling conditions were as follows: 95°C for 7 min, 40 cycles at 95°C for 10 s and Tm for 30 s. A melting curve was obtained to confirm specificity of amplification. Reactions were conducted in 10 µl volumes on 96-well plates containing 1×Dynamo Flash SYBR Green master mix, 0.3 µM of each primer and 1×ROX passive reference dye. Template DNA was used in qPCR reactions in the range 2–12 ng DNA per reaction; a fixed dilution of raw DNA exctract was used. In parallel with the ARGs, the16S rRNA gene copy numbers were quantified.

The qPCR for detecting 16S rRNA gene and ARGs for all the other samples (Assay 2) used the 7900HT Fast Real-Time PCR System (Applied Biosystems). Reactions were conducted in 10 µl volumes on 384-well plates containing 1×Maxima SYBR Green/ROX qPCR Master Mix (Thermo Scientific), 0.4 µM of each primer. The two-step thermal cycling conditions for detecting 16S rRNA gene were as follows: 95°C for 10 min, 40 cycles of 95°C 15 s, 60°C 1 min. For ARGs the thermal cycling conditions were as follows: 95°C for 10 min, 40 cycles at 95°C for 15 s, Tm °C for 30 s and 72°C for 30 s. Melting curves were obtained to confirm specificity of amplification. Template DNA was used in the qPCR reaction in the range 3×10^−4^ to 22.4 ng DNA per reaction. This was obtained by a wider range of dilutions (to avoid inhibition) of the raw DNA extraction product, three levels of 10 fold dilutions were used. Primers and annealing temperature, Tm (°C), are given in Table 1. Number of technical replicates in both qPCR assays was 3.

**Table 1:** Primers used for detecting the target genes and the melting temperatures (Tm) used for primers.

Gene name		Primers				
	Forward	Reverse	Tm °C	Ref.	Amplification efficiency	
					Assay 1	Assay 2
**16S rRNA gene**	5′-AGA GTT TGA TCC TGG CTC AG-3′			[60]		
		5′-CTG CTG CSY CCC GTA GGA-3′	60	[61] modif.		82–100
		5′-CTG CTG CCT CCC GTA GG-3′	60	[61]	87	
**tetC**	5′-TGC GTT GAT GCA ATT TCT ATG C-3′	5′-GGC GCC TAC AAT CCA TG-3′	64	[62]	80	93–101
**tetM**	5′-GCA ATT CTA CTG ATT TCT GC-3′	5′-CTG TTT GAT TAC AAT TTC CGC-3′	60	[61]	90–93	89–108
**sul1**	5′-CGG CGT GGG CTA CCT GAA CG-3′	5′-GCC GAT CGC GTG AAG TTC CG-3′	64	[63]	90–93	81–88
**sul2**	5′-GCG CTC AAG GCA GAT GGC ATT-3′	5′-GCG TTT GAT ACC GGC ACC CGT-3′	64	[62]	102	91–103
**blactx-m-32**	5′-CGT CAC GCT GTT GTT AGG AA-3′	5′-CGC TCA TCA GCA CGA TAA AG-3′	64	[59]	87	89–101
**blashv-34**	5′-GCG TTA TTT TCG CCT GTG TA-3′	5′-AGG TGC TCA TCA TGG GAA AG-3′	60	[63]	92–94	97–108
**blaoxa-58**	5′-GCA ATT GCC TTT TAA ACC TGA-3′	5′-CTG CCT TTT CAA CAA AAC CC-3′	60	[63]	90	97–111

qPCR amplification efficiency is given for 16S RNA gene and for ARGs, R^2^ of the linear range of standards was always >0.99.

### 1.5. Standards used for quantification

A plasmid vector and fragments of ARGs were constructed and used as standards for quantifying the raw qPCR results. The standard plasmids were checked for the correct inserts by sequencing. In Assay 1 for the three Helsinki time points (Dec 2010; March 2011 and June 2011) the 16S rRNA gene quantification standard was genomic DNA from *E. coli* K12 (genome size 4.6 Mbp with seven copies of the rRNA operon). In Assay 2, used for all other samples, the standard for quantifying 16S rRNA gene was constructed from a 16S rRNA gene fragment from the natural aquatic *Chryseobacterium* strain isolated from Emajõgi River, which receives WWTP effluent water, and was cloned into a plasmid and validated by sequencing. Information about the plasmids used and Genbank accession numbers are given in Table 2.

**Table 2:** Plasmids and PCR fragments used as standards.

Gene name	Standard constructs	Accession number	Reference
16S rRNA gene	Assay 1		
	Genomic DNA from *E. coli* K12 genome size 4.6 Mbp with 7 copies of rRNA operon		
	**Assay 2**		
	PCR product cloned in plasmid PGEM-T Easy Vector System (Promega)	KF737394	present study
***tetC***	pDrive (Qiagen)		[62]
***tetM***	pDrive (Qiagen)		[62]
***sul1***	R388		[64]
***sul2***	RSF1010		[64]
***bla_ctx-m-32_***	pUC19	KF737395	present study
***bla_shv-34_***	PGEM-T Easy Vector System (Promega)	KF737397	present study
***bla_oxa-58_***	pUC19	KF737396	present study

### 1.6. Quantification and normalisation of ARGs

Standard curves (Ct per log copy number) for 16S rRNA gene and ARG quantification were obtained for each run using the plasmid constructs (or genomic *E. coli* DNA for 16S rRNA gene in Assay 1) (Table 2) in ten-fold serial dilutions. The gene copy number of a standard was determined from the plasmid/genomic DNA concentration (measured using a NanoDrop in Assay 1 or by fluorescent staining with PicoGreen (Invitrogen) and using VICTOR X3 Multilabel Plate Readers (Perkin-Elmer) in Assay 2). The ARG levels in the sample were calculated using the standard curve equation and measured Ct value, the quality control of raw Ct values for standard curve and unknown samples was done before further analysis. The limit of quantification (LOQ) was defined as the lowest point of the linear part of standard curve: Assay 1, ARGs 100 and 16S rRNA gene 1000 gene copy number per reaction; Assay 2, ARGs and 16S rRNA gene 100, except *sul1* with 10 copy numbers per reaction. The negative control was the reaction mix with nuclease-free water instead of the template DNA. The negative control had always a Ct value at least 3.3 cycles lower than the smallest standard used for calculation of LOQ. Technical replicates were incorporated in the statistical analysis to reflect differences in quantification (see below for details).

### 1.7. Water quality variables

WWTPs in Europe analyse water quality parameters according to the EU directive for urban wastewater treatment 91/271/ECC. These parameters are monitored regularly in accredited laboratories according to standard methods. The parameters used in this study were: biochemical oxygen demand (BOD_7_) - standard method EN 1899-2; total suspended solids (SS) - EN 872; total phosphorus (Ptot) – EN ISO 6878; total nitrogen (Ntot) – EN ISO 11905. In addition, the automated measurements of flow rate and water temperature in the process were recorded. We received the data for water variables from the staff of each WWTP.

### 1.8. Statistics

Linear mixed models were fitted to the data using the functionality of the package lme4 [42]. the statistical software used was R version 3.0.1 [43]. Average levels of gene copy numbers (16S rRNA gene or ARGs) were modelled using the location of the WWTP (Helsinki, Tallinn, Tartu), sample source (IF/EF) and method protocol (Assay1/Assay2) as fixed effects and technical and biological replicates as random effects. Significance of fixed effects was assessed by an F-test using a (pre-specified) significance level of 1%. In addition, the combined effect of each of the environmental variables BOD_7_, SS, Ptot, Ntot, flow rate (i.e water discharge - WD) and temperature in inflowing versus effluent waste on gene copy numbers was described by a linear mixed model version of an analysis of covariance model. Model checking was based on residual plots and normal probability plots using the raw residuals. Models were reduced using the likelihood ratio test. A 1% or 5% significance level was used. Pairwise comparisons were evaluated based on adjusted p-values obtained using the single-step method [44]. Values below LOQ were not included in the analyses.

Principal component analysis (PCA) and its extensions to between groups (BGA) and within groups (WGA) analyses (ade4 package in R) was used to analyse the grouping of inflowing/effluent samples by monitored water quality variables (nutrients, suspended solids, biological oxygen demand, water discharge and temperature).

## Results and Discussion

### 2.1. Selection of ARGs for the study

Initially, we made a screening for 25 selected genes that could spread with higher probability from any WWTPs into environment. Initial selection based on four major criteria: (i) clinically relevant genes (risk to human health) previously detected in WWTPs (e.g. [29]); (ii) genes found in various mobile elements, demonstrating their potential for transfer between bacteria [45]–[48]. (iii) high consumption antibiotics–sulphonamides, tetracyclines, and beta-lactams; (iv) incorporation of long-used antibiotics (tetracycline, sulfonamides) and the newer extended spectrum beta-lactams (carbapenems and 3rd and 4th generation cephalosporins) and their resistance genes. Altogether 12 *bla*, 6 *tet*, 3 *sul*, 3 *qnr* (floroquinolones) and 1 vancomycin resistant gene were tested using traditional PCR. Finally, two resistance genes for sulphonamide (*sul1* and *sul2*), two for tetracycline (*tetM* and *tetC*), and three for extended spectrum beta-lactams (*bla_oxa-58_, bla_shv-34_, and bla_ctx-m-32_*) remained in the study based on their abundance and frequency in screening study. It was necessary to choose a small number of relevant target genes because the variety of different ARGs is large. In the Comprehensive Antibiotic Resistance Database [49] (http://arpcard.mcmaster.ca/accessed Sept. 23, 2013), the number of ARGs is 2153 and testing all these genes quantitatively in one study would be prohibitively difficult.

### 2.2. Abundance of genes (16S rRNA gene and ARGs)

**Figure 1:**
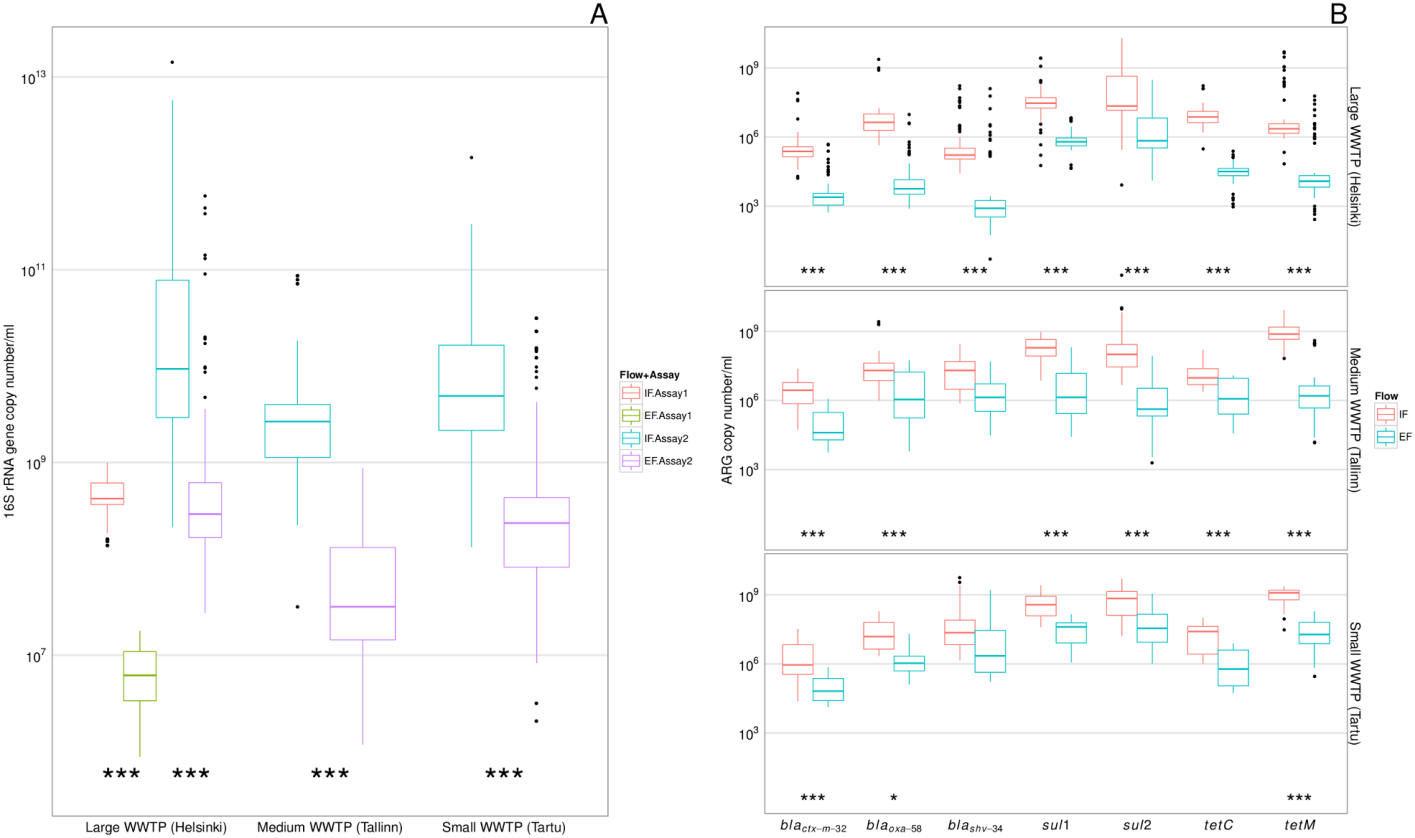
Raw gene copy numbers detected in a WWTP sample (copy number/ml). A - 16S rRNA gene in inflow (IF) and effluent (EF). Assay1 was used only for samples from large WWTP (Helsinki) from Winter 2010 to Autumn 2011; B–Antibiotic resistance genes (ARGs). Statistical significance between inflow wastewater and effluent samples: *** - p<0.01; *0.03>p>0.01. For the pairs not marked the statistical difference between inflow and outflow was statistically insignificant. The line in each box marks the median and boxes: 25th and 75th percentiles; whiskers: 5th and 95th percentiles and outliers ±1.5 * IQR. See Figure S2 in File S1 for abundances of same genes presented by each sampling event.

To evaluate the abundance of the total bacterial community, we quantified 16S rRNA gene in the samples using qPCR. The amplification efficiency is given in Table 1. Initially, the raw gene copy numbers were used to estimate the general changes of bacterial levels during wastewater purification. The copy number of 16S rRNA gene was several orders of magnitude lower in the effluent (EF) than the inflow (IF) (Figure 1A, Table S2 in File S1). The differences between the IF and EF samples were statistically significant in all cities (adjusted p<0.01) (Figure 1A).

The raw gene copy numbers of ARGs/ml decreased during processing in the WWTP water phase. The levels of ARGs detected in the EF were lower than IF in all three plants (Figure 1B, Table S2 in File S1). The decrease of abundance from IF to EF was statistically significant (p<0.01) for all ARGs in the large (Helsinki) WWTP. In the medium (Tallinn) WWTP, for all ARGs except *bla_shv-34_* (p>0.05), the decrease was statistically significant (p<0.01). In the small WWTP (Tartu), the raw abundance of ARGs decreased after purification but the decrease was statistically significant only for *bla_ctx-m-32_* and *tetM* (p<0.01), and weakly significant for *bla_oxa-58_* (p = 0.03). Earlier studies have demonstrated the large variation of treatment plant efficiency in removing microorganisms [50] and micropollutants [51]. which also depends on the capacity of the WWTP plant. Low capacity treatment facilities are more vulnerable to changes in inflowing wastewater composition and flow rates. In addition, the WWTP in Tartu did not have biological post-filtration at the time of sampling.

ARGs are surprisingly rarely quantified directly using community DNA in both IF and EF water samples from WWTPs. As in our study (Figure 1B), a few other studies have found from 100 to 1000 fold reductions of raw ARG copy numbers during the purification process; e.g. *sul1, tetW*; [52]; *tetC, tetA*; [53]. *tetG, tetQ* [54]. In one study, the relative abundance of *sul1* increased, while *sul2* decreased slightly in WWTP effluent [31]. In our study, resistance genes for “older” antibiotics (with exception of *tetC*) were more commonly detected. *sul1*, *sul2,* and *tetM* were present above LOQ in all sites and samples (Table 3 and Figure S2 in File S1). High abundances of various tetracycline resistance genes and sulphonamide resistance genes were also demonstrated in other studies [21], [23]. Quantitative studies that target resistance to the newer beta-lactams in community DNA in WWTPs are almost completely absent–only *bla_TEM_* by Lachmayr et al. [38]. in addition, *bla* genes were quantified in the river water under the influences of wastewater but not directly in the WWTP effluent [55].

**Table 3:** Detection of ARGs in different WWTPs (total of all analyses per gene, n = 15).

ARG	City	IF %(number) detected	EF %(number) detected
***tetC***	Helsinki	93 (14)	80 (12)
	Tallinn	67 (10)	27 (4)
	Tartu	67 (10)	73 (11)
***bla_oxa-58_***	Helsinki	100 (15)	87 (13)
	Tallinn	100 (15)	47 (7)
	Tartu	87 (13)	80 (12)
***bla_shv-34_***	Helsinki	100 (15)	100 (15)
	Tallinn	87 (13)	87 (13)
	Tartu	100 (15)	100 (15)
***bla_ctx-m-32_***	Helsinki	100 (15)	100 (15)
	Tallinn	87 (13)	40 (6)
	Tartu	80 (12)	47 (7)

Only genes that were sometimes not detected are given. sul1, sul2 and tetM were detected 100% in all IF and EF samples from the WWTPs.

### 2.3. Normalised/Relative abundances of ARGs

**Figure 2:**
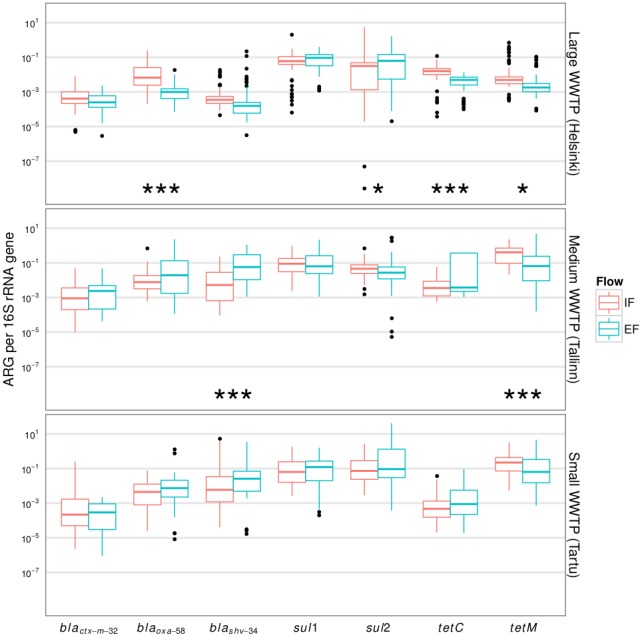
Normalised ARG abundances. Antibiotic resistance gene copy numbers normalised to 16S rRNA gene copy numbers. The results are given for all samples for one gene for a WWTP, no seasonal comparison. Statistically significant comparison results are marked with *** at p<0.01, *0.03>p>0.01. The line in each box marks the median and boxes: 25th and 75th percentiles; whiskers: 5th and 95th percentiles and outliers ±1.5 * IQR.

To avoid inconsistencies among qPCR assays, including sub-optimal efficiency in some cases, we used 16S rRNA gene-normalised values, and the different protocol (Assay1/Assay2) was added as an additional fixed effect into the statistical models. This type of data analysis allows one to quantify the relative changes in ARG abundances, whether more or fewer ARGs appear per microbial genome. When relative abundances were compared, statistically significant (p<0.01) differences between IF and EF were detected in only four cases (Figure 2). For *bla_oxa-58_* and *tet*C in the Helsinki plant we observed a relative decrease after purification. In addition, *tet*M decreased in the Tallinn WWTP EF samples. The only increase in EF was observed for *bla_shv-34_* in the Tallinn WWTP. In all other cases the purification process had no significant effect on the relative abundances of resistance genes.

We conclude from our study that there is neither considerable enrichment (selection) nor purification of ARGs during processes in WWTPs (Figure 2) at the whole community level. Effective selection would be assumed when there are appropriate conditions, i.e. increased concentration of ABs occur. In two WWTPs studied, the measured levels of AB concentrations of several compounds were very low but measurable compared to highly labile beta-lactams [56]. This suggests that conditions could favour enrichment of at least *tet* and *sul* genes within studied WWTPs. Although, quantitative enrichment of ARGs (*sul*) responsible for resistance against refractory ABs with longer history of usage has been demonstrated in some studies [31]. reduction or no change has been observed in most studies [21], [23], [40]. To date, only one study demonstrated similar case of positive selection for newer ARG possessing organisms in a WWTP, which suggests that bacteria harbouring *bla_TEM_* are released more from effluent water compared to wastewater [38].

### 2.4. Abundance of ARGs and treatment efficiency of wastewater

In EU countries, treatment efficiency of WWTPs is estimated by monitoring a few water quality measures, according to European directive 271/1991/EC. The compulsory parameters monitored are total nitrogen (Ntot) and phosphorus (Ptot), Biological Oxygen Demand (BOD_7_), and suspended solids (SS). In addition, a few generic background parameters are measured in all WWTPs e.g. Water Discharge (WD) and temperature. Such water quality parameters are good for evaluating wastewater purification in the traditional sense–removal of excess nutrients, labile organic compounds etc. Obviously, the wastewater was purified of excess nutrients and organic compounds in the WWTPs studied because all parameters were up to an order of magnitude lower in EF than IF. We observed a change of between 6 to 45 fold depending on the parameter. Averages in IF: BOD_7_ - 231; P_tot_ 8; N_tot_ 51 and SS 306 mg/l and in EF: BOD_7_ 5; P_tot_ 0.6; N_tot_ 8 and SS 8 mg/l (Table S1B in File S1). Volumetric concentrations of nutrients, SS and labile organic compounds were higher in the IF of the small (Tartu) than in medium (Tallinn) and large (Helsinki) WWTP (Figure S4 in File S1; IF samples are strongly associated with these variables, permutation test, 1000 replicates, p<0.01). This could be caused by shorter solid retention time in smaller plants [57]. At the same time, the efficiency of purification in the traditional sense did not differ dramatically among plants (Figure S3 in FileS1; residual differences among WWTPs disappear after decomposing the IF/EF level differences, permutation test, 1000 replicates, p>0.05). A relationship between the change of ARG abundances and the efficiency of nutrient removal and temperature has been reported previously (e.g. [58], [59]). These studies suggest that changes in ARG abundance could depend on processes and conditions in the WWTP. However, none of these parameters are designed to estimate threats associated with the spread of either ARB or ARGs. Indeed, in our study, temperature, water discharge and concentrations of nutrients did not help in estimating the efficiency of ARG removal. This was demonstrated by the absence of the combined effect of monitored environmental variables and abundances of ARGs (Figure S4 in File S1). Moreover, a new treatment step (installation of biological post-filtration for final effluent treatment mainly for nitrogen removal) was added during the study period in the Tallinn WWTP (July 2011). However, no statistically significant effect was observed on ARG removal after this event. The Helsinki WWTP had the biological post-filtration installed throughout the study period.

In conclusion, these results force us to reject our original hypothesis. All ARGs were detected in most wastewater and effluent samples, however, the conventional WWTPs under study seem not to be important sites for changes in the relative abundance of ARGs at the whole community level: no enrichment in relative abundance was observed. Furthermore, no additional reduction of ARGs occurred; raw abundance changed in proportion to the decrease of bacterial abundance. We conclude that many unknown factors may influence the biological purification processes in conventional WWTPs and the evaluation of their relationship to ARG removal or selection requires more complex case studies.

## Supporting Information

File S1 **Includes Tables S1 and S2; Figures S1, S2, S3 and S4.**
(PDF)
