# Supplementary material for: Abundances of Tetracycline, Sulphonamide and Beta-Lactam Antibiotic Resistance Genes in Conventional Wastewater Treatment Plants (WWTPs) with Different Waste Load
Source: PLoS One. 2014 Aug 1;9(8):e103705. doi: 10.1371/journal.pone.0103705 (PMC4118896; doi:10.1371/journal.pone.0103705)
Supplement: File S1 — Includes Tables S1 and S2; Figures S1, S2, S3 and S4. (PDF) [file pone.0103705.s001.pdf]

Table S1. A Sampling dates.

Site	Winter 2010	Spring 2011	Summer 2011	Autumn 2011	Winter 2011
**Tallinn**	30.12	22.03	27.06	9.09	8.12
	29.12	23.03	28.06	12.09	12.12
	5.1.11	24.03	30.06	13.09	13.12
**Tartu**	15.12	24.03	15.06	6.09	7.12
	20.12	28.03	17.06	8.09	12.12
	21.12	30.03	20.06	12.09	14.12
**Helsinki**	16.12	25.03	6.06	6.09	8.12
	20.12	28.03	8.06	8.09	12.12
	22.12	30.03	12.06	11.09	14.12

B. Seasonal average of basic parameters. WD -water discharge m^3/day. BOD$_7$- biological oxygene demand, P_{tot} – total phosporus mg/l, N_{tot}- total nitrogen mg/l, SS- susbended solids mg/l.

**WWTP**	**Season**	**WD**	**T°**	**BOD$_7$**			**P_{tot}**			**N_{tot}**	
**SS**				**IF**	**EF**	**IF**	**EF**	**IF**	**EF**	**IF**	**EF**
**Tallinn**	**Winter2010**	103411	9.0	227.0	5.1	6.7	1.2	55.4	12.3	288.7	13.0
**Helsinki**		220411	14.4	314.0	6.2	8.5	0.3	59.1	3.9	314.7	8.7
**Tartu**		28127	7.9	148.0	5.0	5.2	0.5	48.0	10.7	182.7	7.3
**Tallinn**	**Spring 2011**	162710	8.2	140.0	6.1	5.0	1.0	39.8	18.4	298.0	13.3
**Helsinki**		249425	12.4	251.8	4.1	6.9	0.2	51.6	3.9	236.0	6.3
**Tartu**		24516	7.6	111.7	6.6	4.2	0.3	31.3	10.0	263.3	6.0
**Tallinn**	**Summer 2011**	122004	17.1	155.0	1.8	7.4	1.2	43.9	8.2	321.3	6.3
**Helsinki**		217633	16.0	355.4	6.4	8.8	0.3	57.5	3.8	374.7	10.5
**Tartu**		18163	15.5	230.0	6.7	6.3	0.5	52.0	8.3	326.7	10.0
**Tallinn**	**Autumn2011**	136272	17.7	189.0	2.1	7.4	0.9	50.1	4.2	352.0	4.3
**Helsinki**		290612	17.9	206.0	5.2	5.9	0.2	45.3	2.9	248.0	3.2
**Tartu**		14114	15.9	480.0	4.7	21.8	0.7	82.3	9.0	543.3	8.0
**Tallinn**	**Winter 2011**	210607	9.6	81.3	6.1	4.0	0.8	33.2	12.4	244.0	5.0
**Helsinki**		217633	16.0	355.4	6.4	8.8	0.3	57.5	3.8	374.7	10.5
**Tartu**		16186	10.3	228.3	4.0	11.1	0.4	63.3	15.4	236.7	6.7

Table S2. Average (AVG) gene copy number in ml of water. ARGs total is the average value of all the tested ARGs in WWTP. WWTP total is average of the gene in all the plants.

WWTP	Tallinn				Tartu				Helsinki				WWTP total			
	IF		EF		IF		EF		IF		EF		IF		EF	
**Gene**	**AVG**	**SD**	**AVG**	**SD**	**AVG**	**SD**	**AVG**	**SD**	**AVG**	**SD**	**AVG**	**SD**	**AVG**	**SD**	**AVG**	**SD**
ssu rDNA	1 x10^{10}	3 x10^{10}	1 x10^{8}	2 x10^{8}	2 x10^{10}	4 x10^{10}	5 x10^{9}	9 x10^{9}	5 x10^{10}	2 x10^{12}	1 x10^{10}	7 x10^{10}	1 x10^{11}	3 x10^{11}	6 x10^{9}	7 x10^{9}
$\textit{bla}_{ctx32}$	4 x10^{6}	3 x10^{6}	2 x10^{5}	2 x10^{5}	5 x10^{6}	7 x10^{6}	2 x10^{5}	2 x10^{5}	6 x10^{6}	1 x10^{7}	3 x10^{4}	7 x10^{4}	5 x10^{6}	9 x10^{5}	1 x10^{5}	8 x10^{4}
$\textit{bla}_{oxa58}$	2 x10^{8}	3 x10^{8}	9 x10^{6}	2 x10^{7}	4 x10^{7}	3 x10^{7}	3 x10^{6}	3 x10^{6}	1 x10^{8}	2 x10^{8}	5 x10^{5}	1 x10^{6}	1 x10^{8}	7 x10^{7}	7 x10^{6}	6 x10^{6}
$\textit{bla}_{shv34}$	4 x10^{7}	5 x10^{7}	7 x10^{6}	7 x10^{6}	5 x10^{8}	7 x10^{8}	1 x10^{8}	1 x10^{8}	1 x10^{7}	3 x10^{7}	4 x10^{6}	1 x10^{7}	2 x10^{8}	3 x10^{8}	8 x10^{6}	4 x10^{6}
sul1	3 x10^{8}	2 x10^{8}	2 x10^{7}	4 x10^{7}	4 x10^{8}	4 x10^{8}	4 x10^{7}	1 x10^{7}	2 x10^{8}	3 x10^{8}	2 x10^{6}	2 x10^{6}	3 x10^{8}	1 x10^{8}	1 x10^{7}	1 x10^{7}
sul2	8 x10^{8}	1 x10^{9}	1 x10^{7}	2 x10^{7}	1 x10^{9}	1 x10^{9}	2 x10^{8}	2 x10^{8}	1 x10^{9}	2 x10^{9}	2 x10^{7}	3 x10^{7}	10 x10^{8}	2 x10^{8}	2 x10^{7}	7 x10^{6}
tetC	3 x10^{7}	4 x10^{7}	3 x10^{6}	5 x10^{6}	3 x10^{7}	2 x10^{7}	2 x10^{6}	2 x10^{6}	2 x10^{7}	3 x10^{7}	5 x10^{4}	5 x10^{4}	3 x10^{7}	8 x10^{6}	2 x10^{6}	2 x10^{6}
tetM	1 x10^{9}	1 x10^{9}	4 x10^{7}	8 x10^{7}	1 x10^{9}	3 x10^{8}	4 x10^{7}	4 x10^{7}	4 x10^{8}	8 x10^{8}	4 x10^{6}	8 x10^{6}	1 x10^{9}	6 x10^{8}	3 x10^{7}	2 x10^{7}
**ARGs total**	4 x10^{8}	6 x10^{8}	1 x10^{7}	1 x10^{7}	5 x10^{8}	5 x10^{8}	1 x10^{7}	1 x10^{7}	3 x10^{8}	4 x10^{8}	5 x10^{6}	9 x10^{6}	4 x10^{8}	1 x10^{8}	1 x10^{7}	5 x10^{6}

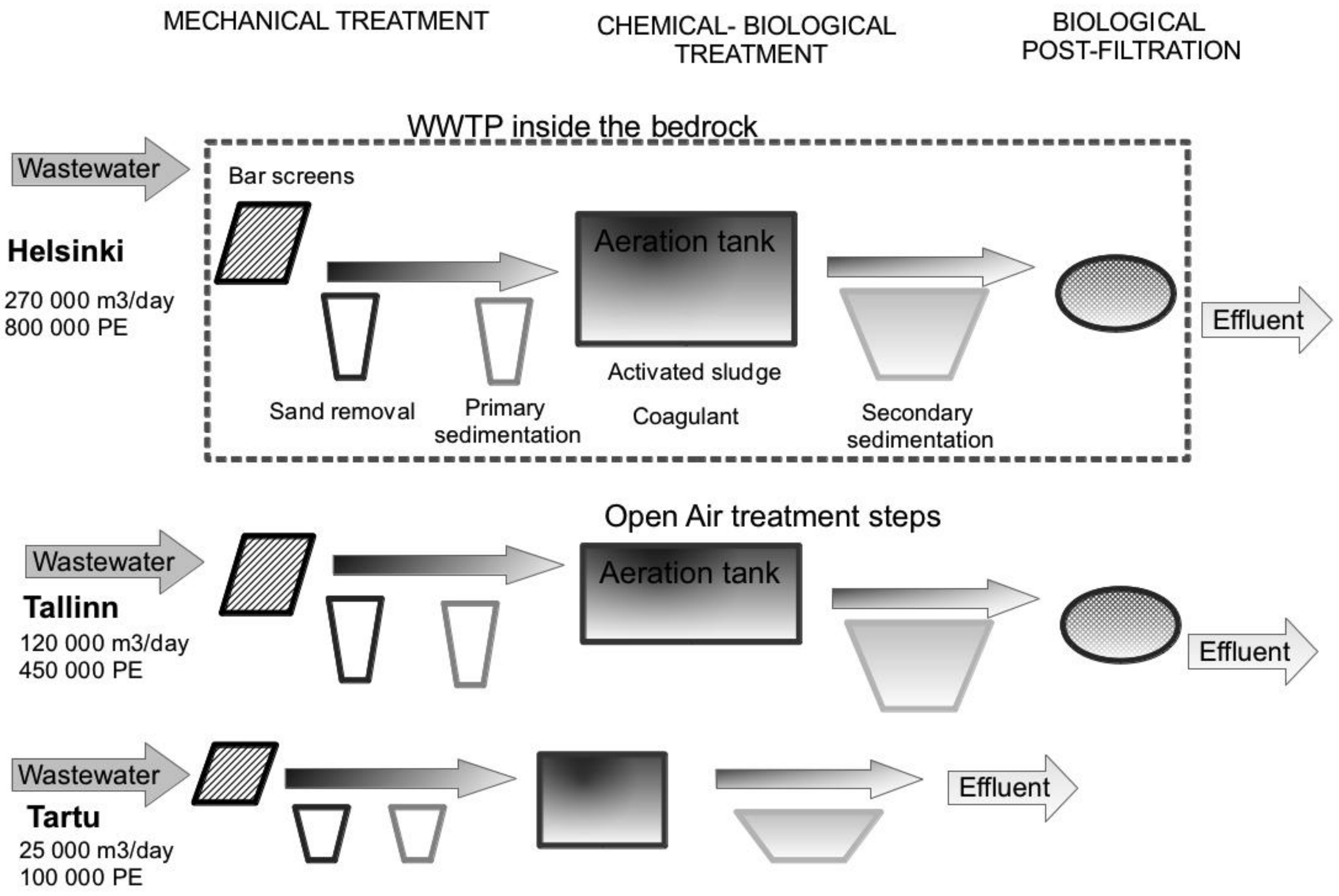

Figure S1. Treatment steps of the studied conventional (three steps: mechanical, biological nad chemical) wastewater treatment plants. Helsinki WWTP is the largest in Nordic countries. Tallinn treats 40% of Estonian wastewater and is the largest WWTP in Estonia. Tartu is representing typical small- size plant in the region. PE- personal equivalent.

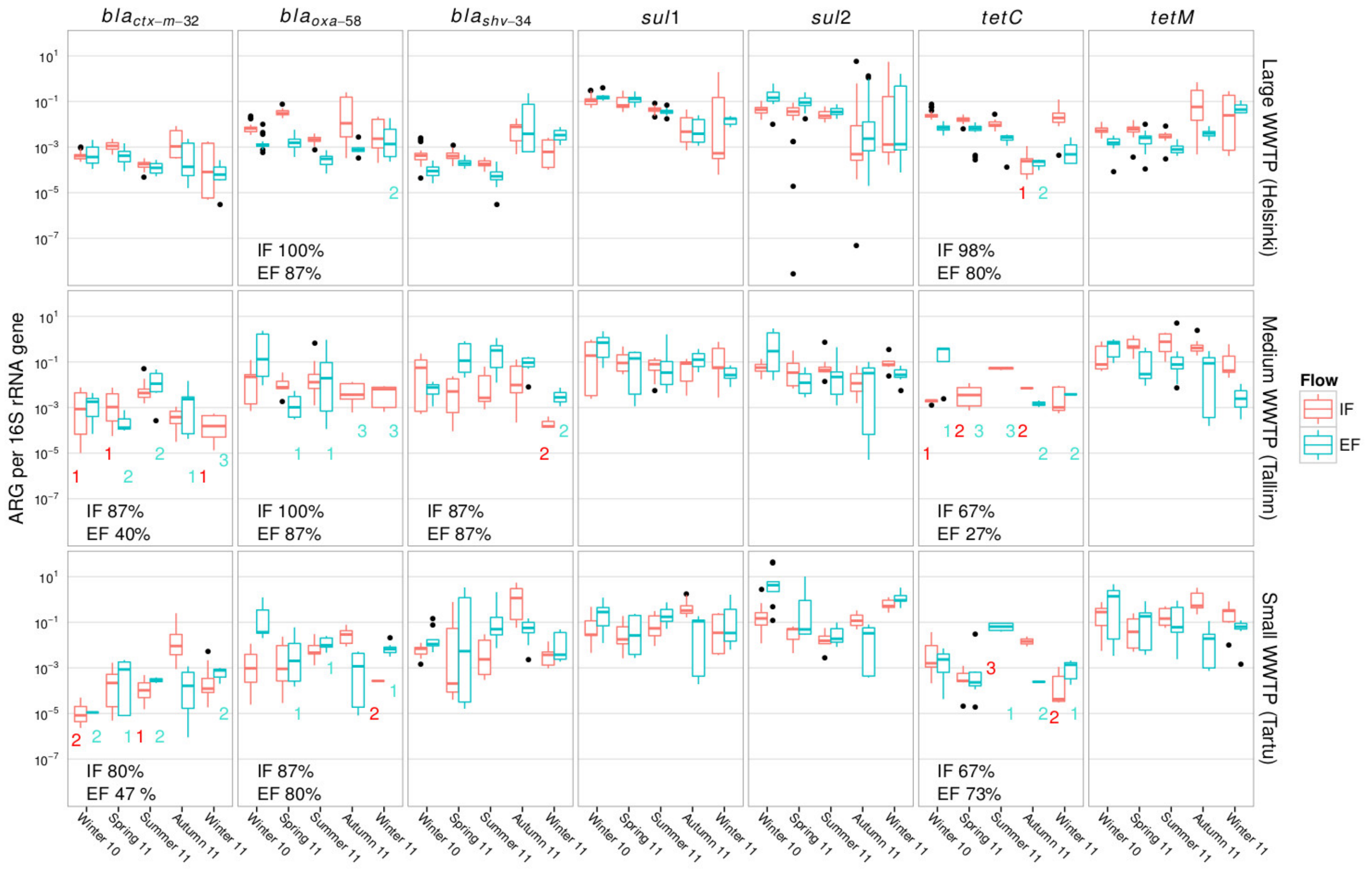

Figure S2. The ssu rDNA normalised values for ARGs are given with ARG detection frequency over the study period. Boxes show the relative abundances of ARGs in WWTPs; ssu rDNA normalised results are given. Numbers on the figure show the number of points (days) not detected in the seasonal sampling set. Detection % of total per ARG tested are given (100% = all 15 test were positive.) For genes where no % values are reported the detection was 100% in IF and EF.

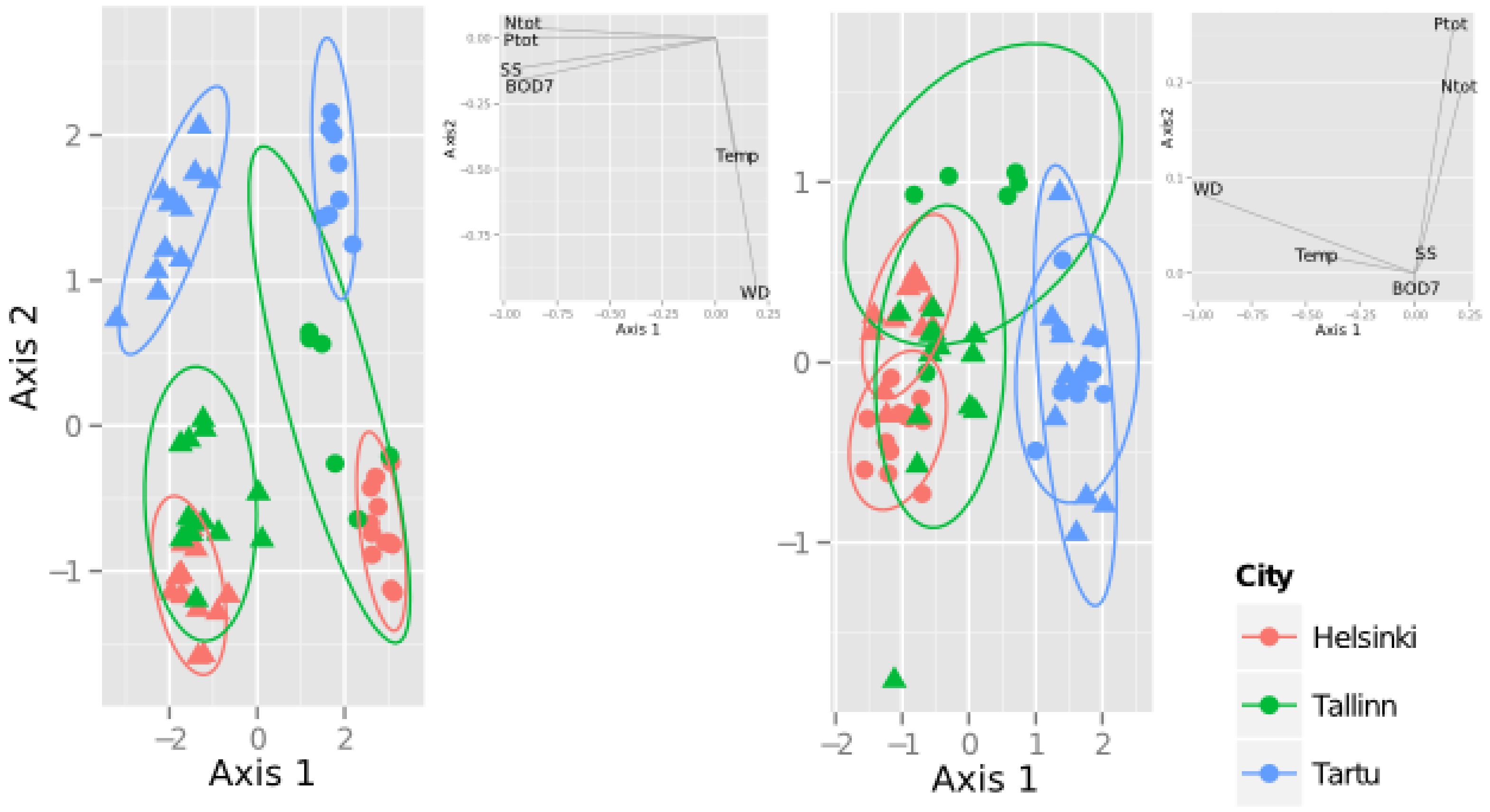

Figure S3. Grouping of effluent (dots) and inflow samples (triangles) according to water quality variables, water discharge (WD) and temperature. Left panel - log-transformed data plotted using between groups analysis to discriminate inflow and effluent samples in various WWTPs, inflowing samples were discriminated from effluents significantly, 1000 replicates, $p < 0.01$. Ellipses indicate the 95% confidence interval around a scatter of points.

Right panel – same samples plotted after decomposing difference in inflow/effluent by within groups analysis. Right upper small panels show correlation with variables (PCA variable scores). Samples from Tartu WTTP were significantly discriminated from the rest of the samples ($p < 0.01$).

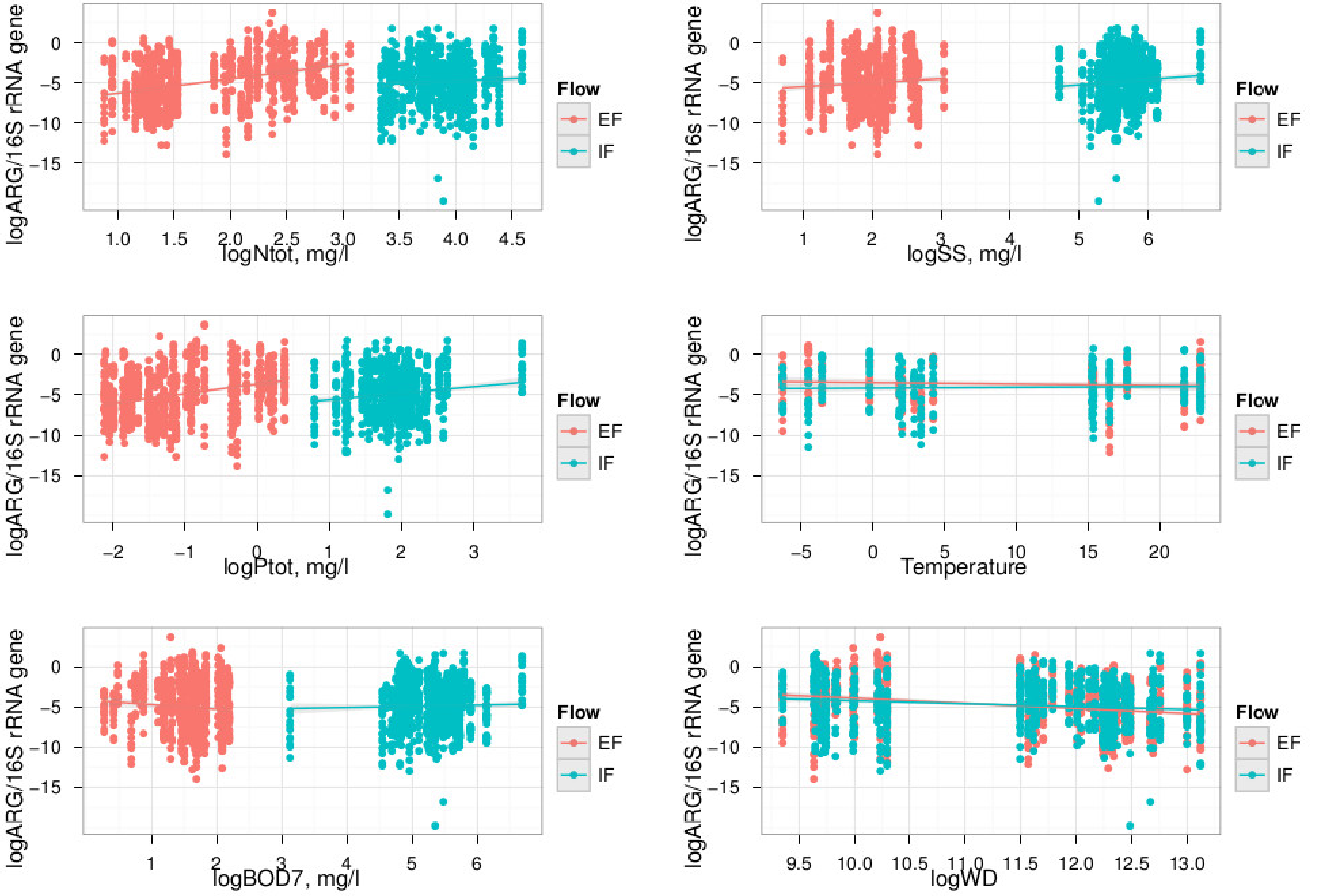

Figure S4. Relationship between relative abundance of ARGs and water quality variables presented as one explanatory variable and response variable (relative ARG abundance). The statistical significance of independent variables in multivariable model: Ntot $p < 0.01$, Ptot $p = 0.23$, BOD7 $p = 0.012$, SS $p < 0.01$, temperature $p < 0.01$, WD $p = 0.02$. Predictive power of model including significant variables (Ntot, SS and temperature: $R^2 = 0.15$.)
